# Synthesis of novel genistein amino acid derivatives and investigation on their interactions with bovine serum albumin by spectroscopy and molecular docking†

**DOI:** 10.1039/c8ra06691d

**Published:** 2018-09-05

**Authors:** Xiaokang Long, Yao-Fu Zeng, Yunmei Liu, Ying Liu, Tangluo Li, Lanqing Liao, Yu Guo

**Affiliations:** Hunan Province Cooperative Innovation Center for Molecular Target New Drug Study Institute of Pharmacy and Pharmacology, University of South China Hengyang 421001 China guoyuhy@126.com

## Abstract

Genistein amino acid derivatives 4a–4d were synthesized and evaluated for their cytotoxic activities against MCF-7, Hela, MGC-803 and HCT-116 cell lines by MTT assays *in vitro*. The results revealed that compounds 4a–4d showed better activity than the parent compound genistein. Particularly, compound 4b displayed the most significant anticancer activity against MGC-803 with an IC_50_ value of 12.08 μM. In addition, the mechanisms of interaction between genistein, compounds 4a–4d and BSA were investigated *via* multi-spectroscopic techniques such as ultraviolet (UV) spectroscopy, fluorescence, circular dichroism (CD), and molecular docking under physiological conditions. The results suggested that endogenous fluorescence of BSA could be quenched by genistein and compounds 4a–4d*via* forming BSA-compound complex, which meant a static quenching mechanism was involved. The negative values of enthalpy (Δ*H*) and entropy (Δ*S*) indicated that interactions between BSA and the ligands were spontaneous, and hydrogen bonding and van der Waals interactions were involved in the BSA-compound complexion formation. The UV, synchronous and 3D fluorescence results revealed that the micro-environment of tryptophan and conformation of BSA were changed after binding to ligands. CD analysis demonstrated the variation in the secondary structure and that the α-helix content of BSA decreased. Eventually, molecular docking was executed to forecast the binding forces and binding sites between BSA and compounds 4a–4d.

## Abstract 2

Introducing amino acid into genistein can not only improve its antitumor activity, but also enhance its binding affinity to BSA.

## Graphical abstract

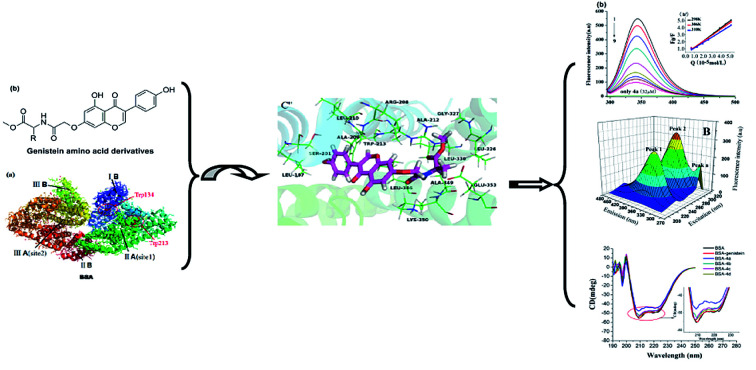

## 1. Introduction

**Fig. 1:**
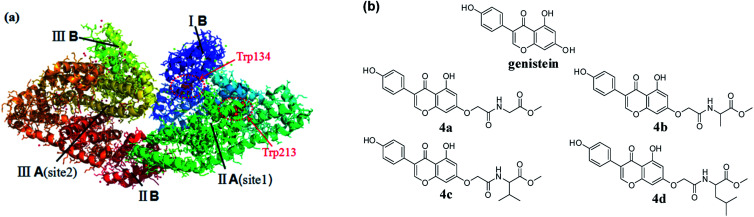
(a) The crystal structure of BSA; (b) the chemical structures of genistein and 4a–4d.

Serum albumins, indispensable parts of the plasma, play vital roles in the absorption, distribution, metabolism, and excretion profiles of various endogenous and exogenous compounds.^1^ Consequently, exploring the interaction between drugs and serum albumins could provide messages for us to interpret the metabolism and transport mechanism of drugs and design new compounds with better biological activities and lower toxicities. Among serum albumins, bovine serum albumin (BSA) is often used as a model for studying the binding of drugs to serum albumin, because it is available and similar to human serum albumin in structure.^2,3^ BSA has three linearly arranged domains (I–III), and each domain consists of A and B sub-domains. Besides, it contains two amino acid residues (tryptophan 134 and tryptophan 212) with endogenous fluorescence.^4,5^ (Fig. 1a).

Genistein, widely existing in soybean, possesses a broad range of pharmacological activities such as antitumor, antioxidant, antiseptic, anti-osteoporotic and so on.^6–9^ However, its poor solubility and low bioavailability limited its potential in clinical treatment. Amino acids are the basic components of protein and participate in a variety of physiological activities in the body. The introduction of amino acids into drugs as amino acid prodrugs has become a popular strategy for scholars. A large number of experimental data showed that amino acid prodrugs can improve solubility, permeability as well as metabolic stability of the parent substances.^10–12^ In recent years, the interactions between chlorinated,^13^ alkylated and trifluoromethylated genisteins and BSA were reported by several groups.^14^ However, the studies on the structural affinity relationship between genistein amino acid derivatives and BSA have not yet been published.

Herein four genistein conjugates modified with amino acid 4a–4d (Fig. 1b) were synthesized and evaluated as anti-cancer agents. Besides, the mechanisms of interaction between genistein amino acid derivatives 4a–4d and BSA were explored using multi-spectroscopy (UV, synchronous fluorescence, 3D fluorescence, CD spectroscopy) and molecular docking.

## 2. Materials and methods

### 2.1 Reagent and apparatus

Genistein (≥97%) and BSA (≥99%) were obtained from Aladdin's Reagent Co., Ltd. (Shanghai, China). Tris(hydroxymethyl) aminomethane (Tris) (≥99%) was obtained from Solarbio Science & Technology Co., Ltd. (Beijing, China). Compounds 4a–4d (≥97%) were synthesized in the laboratory in University of South China.

The stock solution of BSA (10 μM) was prepared using 0.05 mol L^−1^ Tris–HCl buffer solution containing 0.05 mol L^−1^ NaCl (pH = 7.4). The stock solutions of genistein and its amino acid derivatives 4a–4d (1000 μM) were dissolved in methanol. All reactants and solvents used in synthesis were analytically pure and the distilled water was used in experiments.

### 2.2. Synthesis of genistein amino acid methyl ester derivatives

**Fig. 2:**
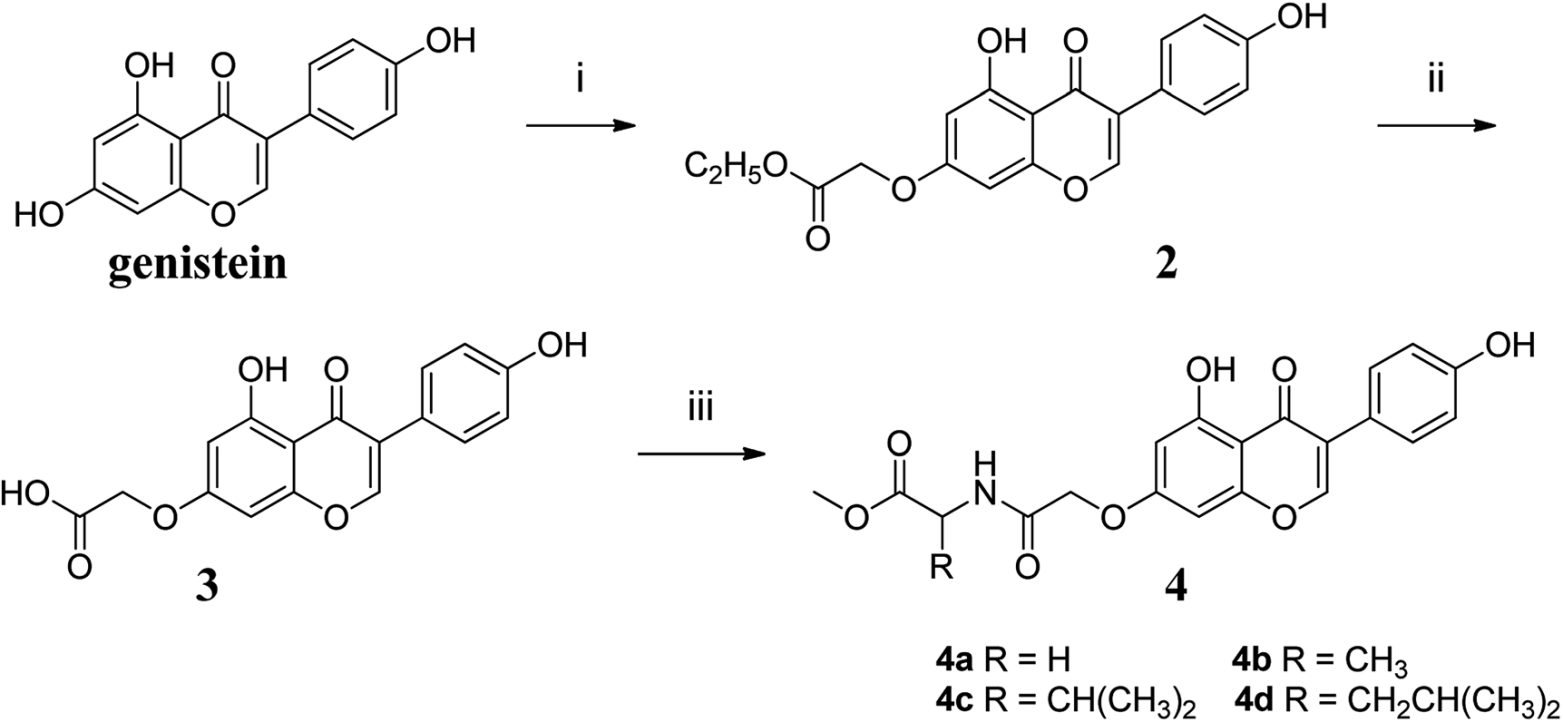
Reagents and conditions: (i) KOH, DMF, 60 °C, 1 h, then BrCH_2_COOC_2_H_5_, 60 °C, 5 h; (ii) CH_3_OH, H_2_O, KOH (1 mol L^−1^), 60 °C, 0.5 h, then H_2_SO_4_ (0.5 mol L^−1^), at room temperature, 0.5 h; (iii) EDCI, HOBT, DMF, 0 °C, 4 h, then DIPEA, DMAP, amino acid methyl ester, r.t., 24 h.

The synthetic route to compounds 4a–4d was shown in Fig. 2. The detailed procedure and characterization spectroscopy of the compounds could be found in the ESI.†

### 2.3 Cytotoxicity assays

#### 2.3.1 Cell culture

All cell lines were purchased from the Cell Bank of Type Culture Collection of Chinese Academy of Sciences (Shanghai, China). MGC-803 was routinely cultured in RPMI-1640 medium containing 10% FBS and 1% penicillin/streptomycin. MCF-7, HCT-116 and Hela were routinely cultured in medium DMEM/high glucose containing 10% FBS and 1% penicillin/streptomycin. All cell lines were incubated in culture at 37 °C in air with 5% CO_2_ for 24 h.

#### 2.3.2 MTT assays

All cell lines were plated in 96-well plates, and each well contains 4000 cells in 100 μL culture medium. Then the cells were treated with genistein or its amino acid derivatives dissolved in DMSO at different doses in a CO_2_ incubator at 37 °C for 48 h, and DMSO was used as vehicle control. After removing the culture medium, 10 μL MTT and 90 μL blank culture medium were added to each well and incubated for additional 4 h at 37 °C. The absorbance was determined by a microplate-reader at 490 nm.

### 2.4 Fluorescence spectra measurements

#### 2.4.1 Fluorescence quenching spectroscopy

Fluorescence spectra were carried on a F-7000 fluorescence spectrophotometer (Japan) fitted out with a 1.0 cm quartz dish. The excitation wavelength was set at 280 nm with 5/5 nm slit widths, while the emission wavelength ranged from 290 nm to 500 nm at different temperatures. The BSA solution (1 μM) was titrated with genistein and its amino acid derivatives 4a–4d for three times. The final concentrations of genistein and 4a–4d were (0.0, 6.4, 12.8, 19.2, 25.6, 32, 38.4, 44.8, and 51.2) μM.

#### 2.4.2 Synchronous fluorescence spectroscopy

The synchronous fluorescence spectra of free BSA and BSA-compounds system were recorded at the emission wavelength from 240 nm to 400 nm at 298 K for three times. The scanning intervals were set to Δ*λ* = 15 nm and Δ*λ* = 60 nm (Δ*λ* = Δ*λ*_em_ − Δ*λ*_ex_), respectively. Other parameters were the same as Section 2.4.1.

#### 2.4.3 Three-dimensional fluorescence spectroscopy

The 3D fluorescence spectra of genistein and compounds 4a–4d were recorded with scanning excitation wavelength ranged from 200 nm to 300 nm with 10 nm interval, and emission wavelength ranged from 290 nm to 470 nm with 5 nm interval for three times. Other parameters were the same as Section 2.4.1.

### 2.5 Ultraviolet-visible absorption spectroscopy

The UV spectra were carried on a UV-2450 UV-vis spectrophotometer (Shimadzu, Japan) in the wavelength range of 200–350 nm at 298 K for three times. The BSA solutions (1 μM) with and without genistein and compounds 4a–4d (1 μM) were measured. The Tris–HCl buffer solution was used as the blank control.

### 2.6 Circular dichroism spectroscopy

The CD spectra of BSA-genistein and BSA-compounds 4a–4d were performed in the range of 190–250 nm with a scan speed of 100 nm min^−1^ on a J-1500 spectrophotometer (Applied Photo-physics Ltd, Surrey, UK) with a 1 mm quartz dish at 298 K. The concentrations of BSA and compounds 4a–4d were 8 μM. Spectra were recorded with the resolution of 1 nm, bandwidth of 1 nm and response of 1 s.

### 2.7 Molecular docking

The X-ray crystal structure of BSA (ID: 4JK4) was got from the Protein Data Bank. The original ligand of the BSA structure was extracted and the water molecule of BSA was removed and hydrogen was added. Atomic charges of BSA were reckoned using Gasteiger–Huckel and AMBER7FF99 method.^14^ Then the ‘protomol’ file of BSA was generated.

The structures of genistein and its derivatives 4a–4d were optimized by minimization with the minimum RMS of 0.001 using MM2 method implemented by Chem3D Pro 14.0 software.^15^ Then, it was further optimized using Tripos force field and Gasteiger–Huckel methods. Finally, the molecular docking was measured through the Surflex-Dock module in SYBYL-X2.0 software.^16^ The docked conformation was visualized using PyMol.^17^

## 3. Results and discussions

### 3.1 Cytotoxic assays

Genistein and its amino acid derivatives 4a–4d were screened for their cytotoxic activities against four human cancer cell lines (MCF-7, Hela, MGC-803 and HCT-116). The results were summarized in Table 1. As we can see in Table 1, compounds 4a–4d displayed stronger activities than the parent compound genistein, which meant introducing amino acid into genistein was beneficial to improve its anticancer activity. Particularly, compound 4b bearing alanine chain showed the best cytotoxic activity against MGC-803 cell lines with IC_50_ value of 12.08 μM.

**Table 1:** Cytotoxic activities of genistein and its amino acid derivatives 4a–4d

Compounds	Cancer cell lines (IC_50_, μM)^a^			
	MCF-7	Hela	MGC-803	HCT-116
4a	>100	66.63 ± 3.60	24.35 ± 1.27	65.28 ± 2.91
4b	21.32 ± 2.24	90.27 ± 0.47	12.08 ± 0.06	>100
4c	99.73 ± 0.11	82.71 ± 0.15	33.53 ± 0.13	42.64 ± 0.15
4d	47.00 ± 3.53	68.14 ± 1.05	35.35 ± 3.72	92.87 ± 0.04
Genistein	>100	>100	>100	>100

a Each value represents mean ± SD of three experiments.

### 3.2 Fluorescence quenching of BSA

**Fig. 3:**
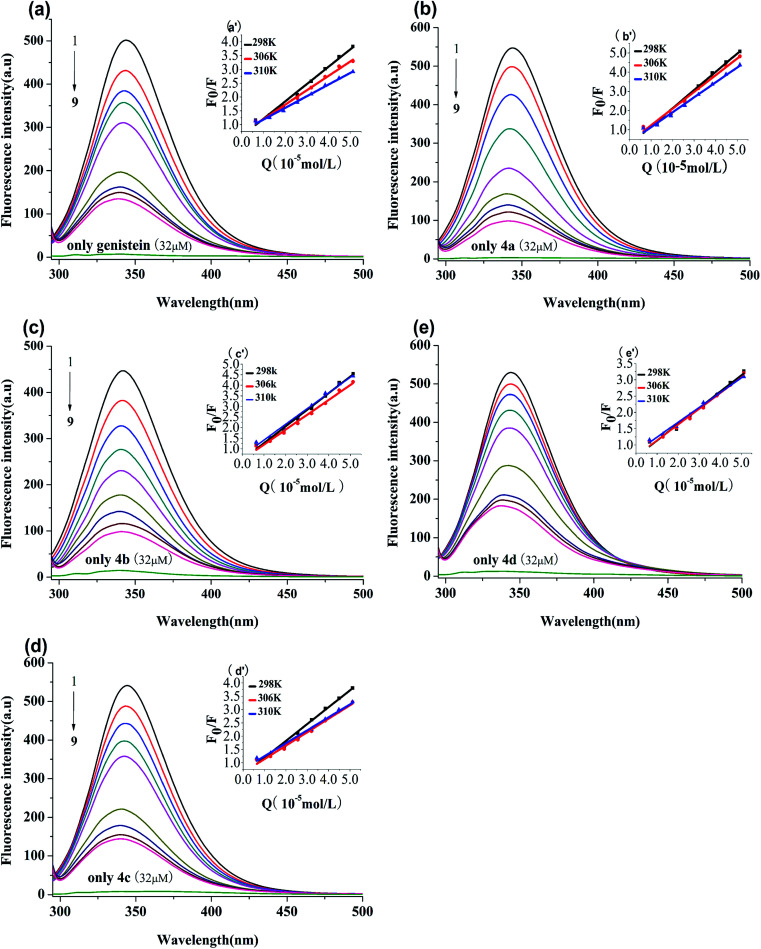
Fluorescence spectra of BSA (1 μM) solution in the presence of genistein (a), 4a (b), 4b (c), 4c (d), 4d (e), at *λ*_ex_ = 280 nm at 298 K. The concentrations of all compounds were from 0 to 51.2 μM at intervals of 6.4 μM. Inset: Stern–Volmer plots of genistein (a′), 4a (b′), 4b (c′), 4c (d′), 4d (e′).

BSA has endogenous fluorescence, because it possesses tryptophan, tyrosine and phenylalanine residues.^18^ The tryptophan residue is the most important factor in the production of intrinsic fluorescence, which is frequently used as a probe to explore the interaction between drugs and BSA.^15^ In our study, the binding process between genistein, compounds 4a–4d and BSA were investigated *via* fluorescence spectroscopy at three unequal temperatures. As shown in Fig. 3, BSA had a strong emission peak at around 347 nm when excited at 280 nm, while the fluorescence absorption of test compounds alone were negligible. With increasing concentrations of genistein and 4a–4d (from 0 to 51.2 μM), the fluorescence intensity of BSA decreased dramatically. Besides, a blue shift (>3 nm) of the maximum emission wavelength (*λ*_em_) appeared, which indicated that genistein and its analogs may bind to BSA, change the micro-environment of tryptophan residue and then quench intrinsic fluorescence of BSA.

### 3.3 Quenching mechanism investigation

#### 3.3.1 Quenching type

The type of fluorescence quenching is usually divided into dynamic and static quenching, which can be differentiated from temperature, viscosity and collision rate constant. With the increase of temperature, the static quenching constants decrease owing to the destabilization of the ground-state complex at higher temperature. In contrast, the dynamic quenching constants increase with the rise of temperature, because the molecular diffusion coefficient becomes larger.^15^

The Stern–Volmer equation is often applied to reveal the fluorescence quenching mechanism:^19^1
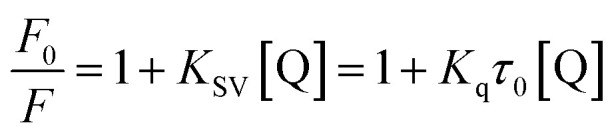
where *F*_0_ and *F* are the fluorescence intensities of complex with and without of quencher, respectively.^18^ [Q] denotes the concentration of quencher. *K*_sv_ and *K*_q_ are quenching constant and quenching rate constant of BSA, respectively.^20^*τ*_0_ is the lifetime of fluorophore in the absence of genistein, 4a–4d and equals to 10^−8^ s.^21^ The inset of Fig. 3 showed the Stern–Volmer plots of compounds-BSA system, which displayed good linear relationship. It can be observed that the *K*_sv_ values decreased gradually with rise in temperature in Table 2. Furthermore, *K*_q_ values were in the order of 10^12^, which were larger than the maximum diffusion collisional rate constant (2.0 × 10^10^ M^−1^ s^−1^).^22^ Hence, we propose that the quenching mechanism of BSA fluorescence was a ground-state complex formed rather than dynamic collision.

**Table 2:** Quenching constants induced by genistein and compounds 4a–4d at different temperatures

Compounds	*T* (K)	*K*_SV_ (10^4^ M^−1^)	*K*_q_ (10^12^ M^−1^ s^−1^)	*r* ^2^ a
Genistein	298	6.2416 ± 0.0984	6.2416	0.9902
	306	5.3472 ± 0.1242	5.3472	0.9911
	310	4.3285 ± 0.1005	4.3285	0.9924
4a	298	9.3690 ± 0.1055	9.3690	0.9900
	306	9.0645 ± 0.0720	9.0645	0.9904
	310	7.7362 ± 0.1223	7.7362	0.9903
4b	298	8.0160 ± 0.1379	8.0160	0.9922
	306	6.9409 ± 0.0956	6.9409	0.9913
	310	7.6019 ± 0.1604	7.6019	0.9924
4c	298	6.2575 ± 0.1717	6.2575	0.9904
	306	5.0010 ± 0.1067	5.0010	0.9906
	310	4.8646 ± 0.1370	4.8646	0.9918
4d	298	4.9339 ± 0.0500	4.9339	0.9896
	306	4.8535 ± 0.0465	4.8535	0.9891
	310	4.5804 ± 0.0360	4.5804	0.9922

a *r* ^2^ is the correlation coefficient.

#### 3.3.2 Binding parameters

Double-logarithmic eqn (2) is often used to explore the binding parameters of static quenching, such as binding constant (*K*_b_) and binding sites (*n*):^23^2
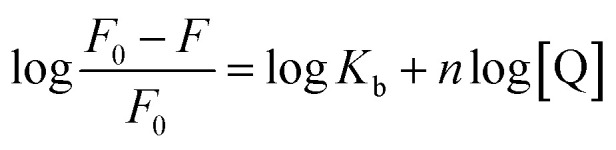


**Fig. 4:**
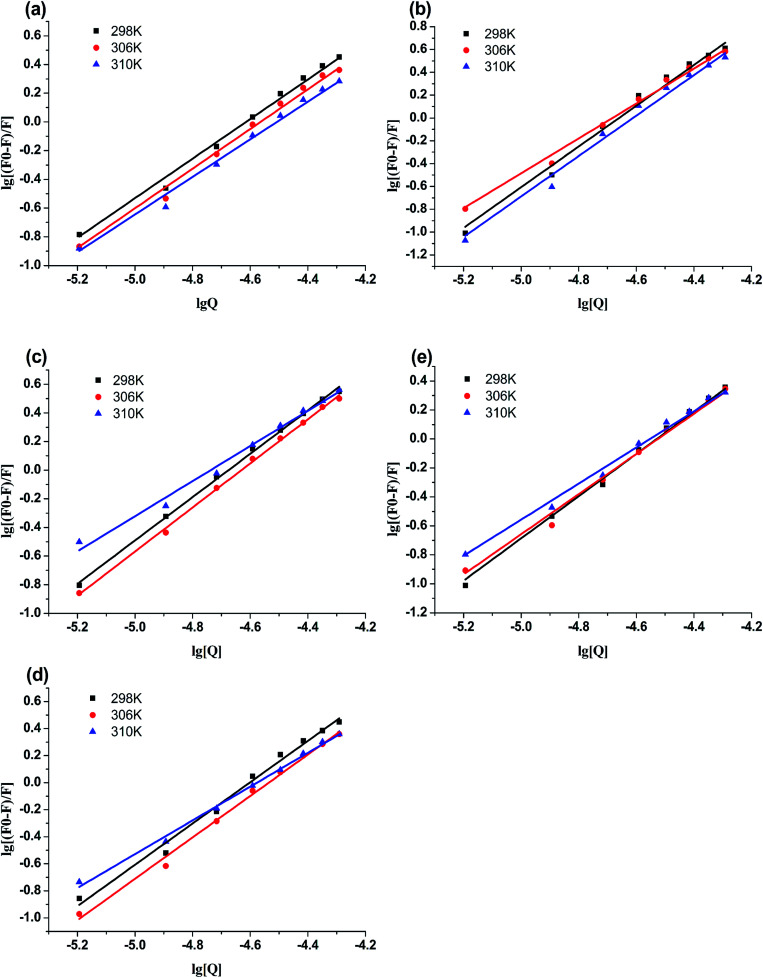
Double-log plots of genistein (a), 4a (b), 4b (c), 4c (d) or 4d (e) binding to BSA at different temperatures.

The plots of log[(*F*_0_ − *F*)/*F*_0_] *versus* log[Q] were displayed in Fig. 4. The values of *K*_b_ and *n* for compounds-BSA system were recorded in Table 3. According to Table 3, all the number of binding sites were more than 1, which meant there was not only a strongly single binding site between compounds 4a–4d and BSA.^24^ The binding constants (*K*_b_) decreased with rise in temperature, which were consistent with the trends of *K*_sv_. Furthermore, the binding constants of compounds-BSA increased in the following order: 4a > 4b > 4c > 4d > genistein, which meant the introduction of amino acid into genistein can enhance its interaction with BSA. And the larger the alkyl chain of amino acid, the lower affinity to BSA. The reasons for this phenomenon are as follows: introducing amino acid into genistein can enhance its hydrophobicity, which was beneficial to penetrate into the hydrophobic tryptophan residues of BSA. Besides, the amide group of compounds 4a–4d can form N–H–O or O–H–S or N–H–S type of hydrogen bonds with amino groups, hydroxyl groups and sulfhydryl groups on the surface of BSA. On the other side, with the increase of the length of the alkyl chain of compounds 4a–4d, the effects of steric hindrance became stronger, which weakened their capacities to bind with BSA.

**Table 3:** The binding and thermodynamic parameters for genistein and its derivatives binding to BSA

Compounds	*T* (K)	log *K*_b_	*K*_b_ (M^−1^)	*n*	*R* ^2^ a	Δ*H* (kJ M^−1^)	Δ*S* (J K^−1^ M^−1^)	Δ*G*_exp_^b^ (kJ M^−1^)
Genistein	298	6.58 ± 0.13	3.90 × 10^6^	1.42	0.9933	−59.09	−71.83	−37.69
	306	6.36 ± 0.16	2.41 × 10^6^	1.39	0.9930			−37.11
	310	6.17 ± 0.05	1.49 × 10^6^	1.37	0.9920			−36.83
4a	298	8.64 ± 0.04	4.35 × 10^8^	1.86	0.9909	−123.52	−249.37	−49.21
	306	8.02 ± 0.06	1.05 × 10^8^	1.72	0.9923			−47.21
	310	7.81 ± 0.08	6.52 × 10^7^	1.66	0.9904			−46.22
4b	298	7.17 ± 0.14	1.54 × 10^7^	1.86	0.9909	−175.21	−446.98	−42.01
	306	7.10 ± 0.03	1.26 × 10^7^	1.53	0.9980			−38.43
	310	5.81 ± 0.08	6.46 × 10^5^	1.23	0.9896			−36.65
4c	298	7.07 ± 0.10	1.19 × 10^7^	1.54	0.9919	−177.34	−456.55	−41.29
	306	6.92 ± 0.03	8.38 × 10^6^	1.53	0.9947			−37.63
	310	5.69 ± 0.05	4.97 × 10^5^	1.24	0.9958			−35.81
4d	298	6.80 ± 0.19	6.65 × 10^6^	1.50	0.9977	−126.67	−292.42	−39.53
	306	6.66 ± 0.15	4.78 × 10^6^	1.47	0.9958			−37.19
	310	5.84 ± 0.07	7.04 × 10^5^	1.28	0.9937			−36.02

a *R* ^2^ is the correlation coefficient.

b Δ*G*_exp_ = Δ*H* − *T*Δ*S*.

#### 3.3.3 Binding forces to BSA

To clarify the binding forces between genistein, 4a–4d and BSA, the thermodynamic parameters were studied *via* the van't Hoff equations:^25^3
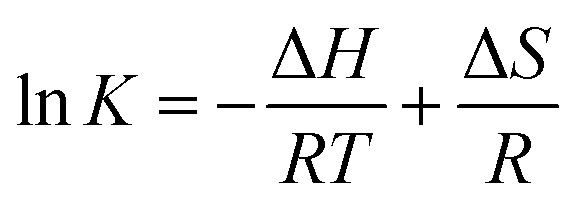

4 Δ*G* = −*RT* ln *K* = Δ*H* − *T*Δ*S*

where *T* denotes the experimental temperature, *K* denotes the binding constant at the corresponding temperature, *R* is the gas constant.^26^ According to Ross and Subramanian's previous research,^27^ the characteristic for van der Waals interactions or/and hydrogen bonding is Δ*H* < 0 and Δ*S* < 0, for hydrophobic interaction is Δ*H* > 0 and Δ*S* > 0, and for electrostatic interactions is Δ*H* ≈ 0 and Δ*S* > 0.^28^ The calculation results of the thermodynamic parameters were recorded in Table 3. As shown in Table 3, the Δ*G* < 0 meant that the interactions between genistein, compounds 4a–4d and BSA were spontaneous. The Δ*H* < 0 and Δ*S* < 0 suggested that van der Waals or hydrogen bonding was the main force in the process of binding compounds 4a–4d to BSA.

### 3.4 Conformation investigation

#### 3.4.1 Synchronous fluorescence spectroscopy

**Fig. 5:**
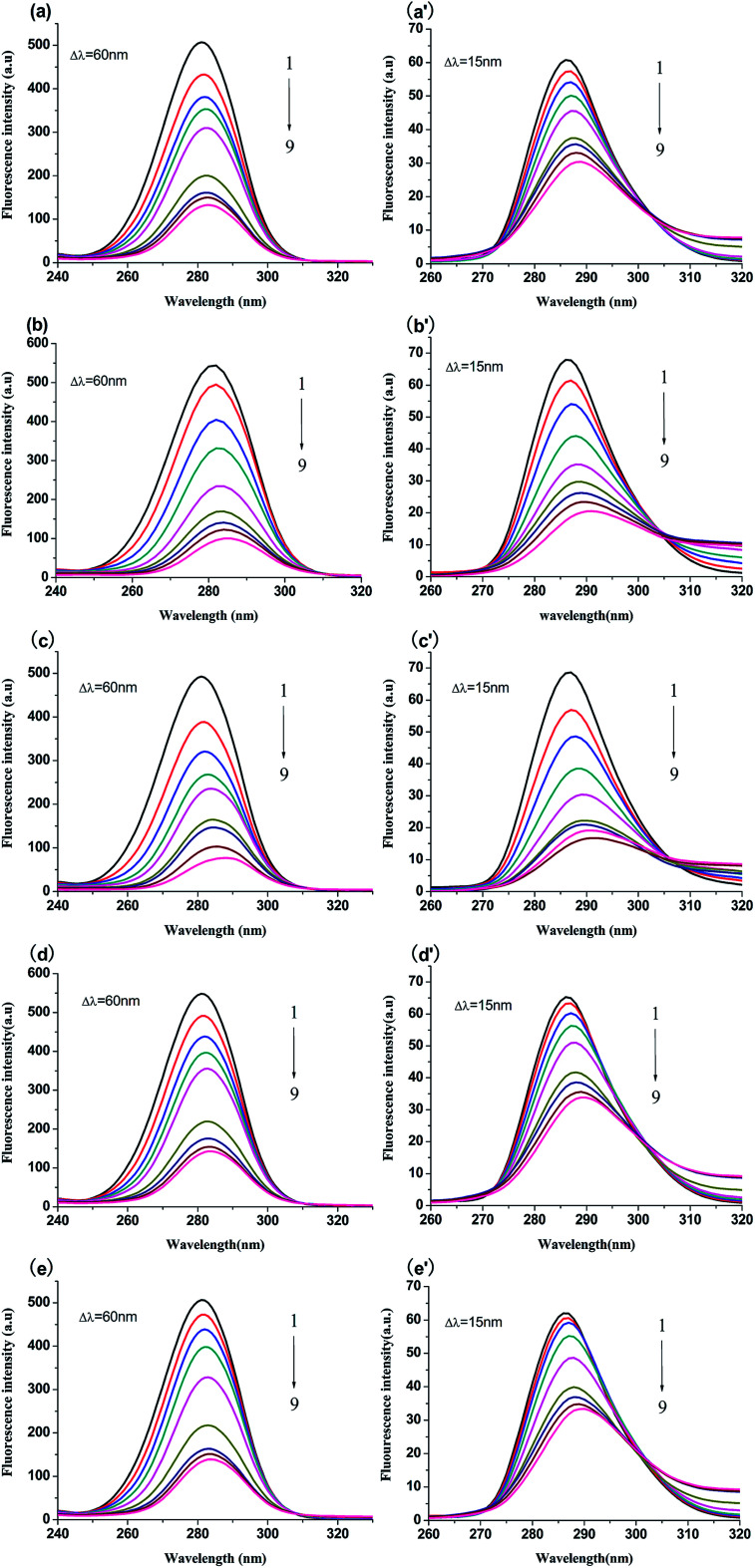
Synchronous fluorescence spectra of BSA (1 μM) with and without genistein, 4a, 4b, 4c or 4d at 298 K when Δ*λ* = 60 nm (a–e) and Δ*λ* = 15 nm (a′–e′). The concentrations of all compounds from 0 to 51.2 μM at intervals of 6.4 μM.

The micro-environment of Trp and Tyr residues of BSA is often studied by synchronous fluorescence spectroscopy. When the intervals of wavelength of synchronous fluorescence spectra are equal to 15 nm and 60 nm, which are characteristics of Tyr and Trp residues, respectively.^29^ Generally, the shift of maximum emission wavelength is used to represent the change of the polarity of the micro-environment surrounding Tyr or Trp residues.^30^ The red shift suggests that the polarity surrounding Tyr or Trp residues increases and hydrophobicity decreases. In reverse, the blue shift means the hydrophobicity increases and the polarity decreases.^31^ The synchronous fluorescence spectra of compounds-BSA are exhibited in Fig. 5. As shown in Fig. 5, it was apparent that the fluorescence intensities of Trp and Try residues both decreased along with a red shift of the emission peak, which indicated that the hydrophobicity around Trp and Tyr residues decreased. Moreover, the level of red shift for Typ residues (>4.0 nm) was greater compared to that for Trp residues (>2.5 nm), indicating that the micro-environment around Typ residues became less hydrophobic.

#### 3.4.2 UV-vis absorption spectroscopy

**Fig. 6:**
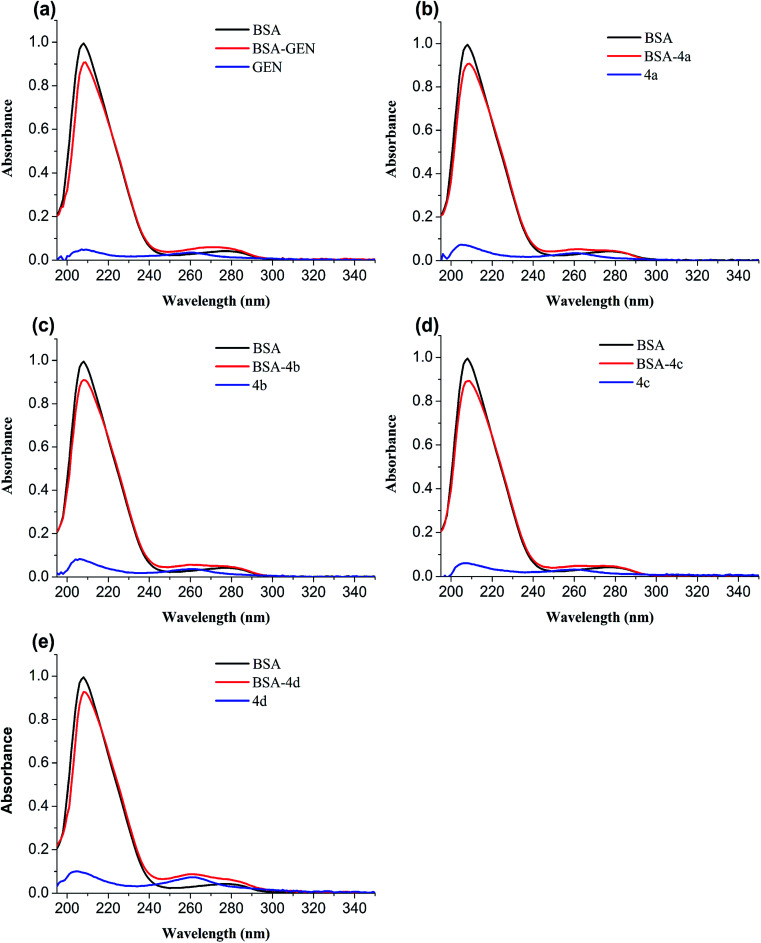
UV absorption spectra of BSA in the absence and presence of genistein (a), 4a (b), 4b (c), 4c (d), or 4d (e). The concentrations of BSA and compounds 4a–4d were 1 μM.

The conformational change of protein caused by binding to the ligand is usually studied by UV-vis spectroscopy. In order to verify the conformational change, the UV-absorption spectra of free BSA and BSA-compounds system are implemented. As shown in Fig. 6, BSA has two absorption peaks: the strong peak around 208 nm reflected the framework of protein and the peak around 280 nm ascribed to absorption of Trp, Tyr, and Phe residues.^32^ The absorbance of the strong peak decreased with addition of genistein and 4a–4d along with a red shift, showing that BSA-compounds complex were formed. They could reduce the α-helical content and change the conformation of BSA.^33^

#### 3.4.3 3D fluorescence spectra

**Fig. 7:**
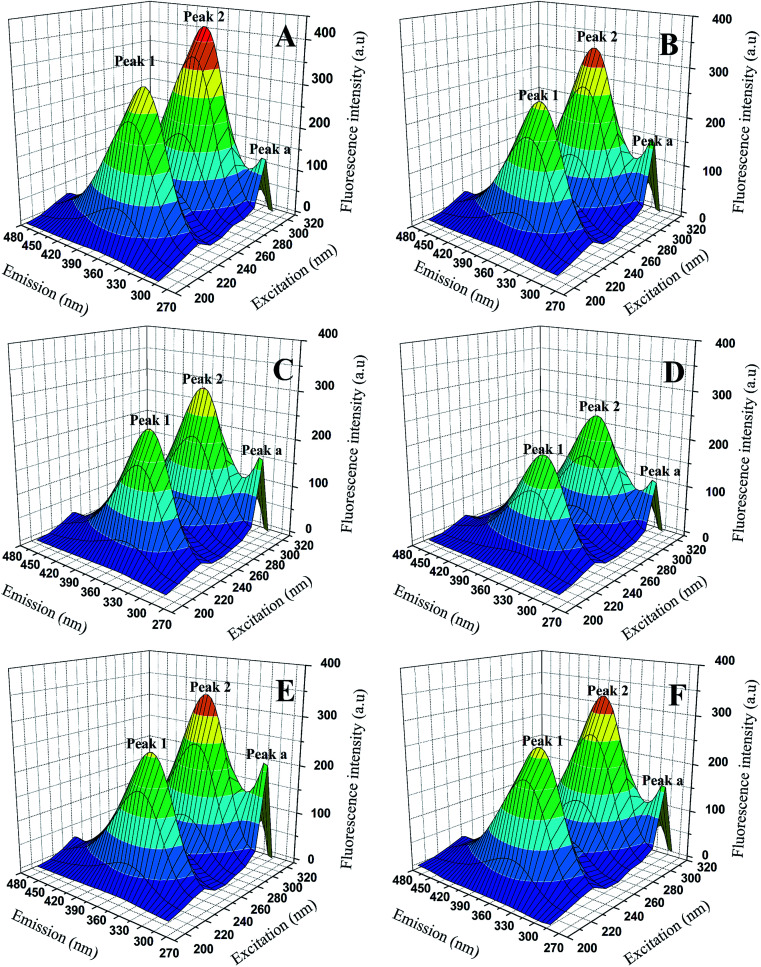
3D fluorescence spectra of BSA (A) and BSA-genistein (B), BSA-4a (C), BSA-4b (D), BSA-4c (E), BSA-4d (F). *C*_(compounds)_ = 3.0 μM, *C*_(BSA)_ = 1.0 μM.

Three-dimensional (3D) fluorescence spectrum can show us more intuitive information about the micro-environmental changes of endogenous fluorophores (Trp, Typ, Phe) and conformational changes of proteins when binding to the ligands. The 3D fluorescence spectrum of BSA and BSA-compounds system were showed in Fig. 7 and the data were summarized in Table 4. As we can see, three peaks were observed in the 3D fluorescence spectra. Peak a (*λ*_ex_ = *λ*_em_) was the Rayleigh scattering peak and peak 1 corresponded to the characteristic peak of polypeptide backbone structure due to π → π* transition, while peak 2 represented the spectral feature of tryptophan and tyrosine residues in protein.^25,34,35^ As shown in Fig. 7, with the addition of genistein and compounds 4a–4d, the intensity of emission peak a increased, which indicating the diameter of BSA increased. In contrast, the fluorescence intensities of emission peak 1 and emission peak 2 decreased dramatically along with a slight red shift (∼2 nm) of peak 2, revealing that the conformation of the peptide backbone was changed and the polarity surrounding endogenous fluorophore (Trp and Typ residues) of BSA increased. Detailed data were recorded in Table 4.

**Table 4:** 3D fluorescence spectral parameters of BSA with and without genistein or 4a–4d at 298 K

System	Peak no.	Position *λ*_ex_/*λ*_em_ (nm/nm)	Intensity (a.u)
[BSA]	1	230/355	326.1
	2	280/353	422.7
	a	290/295	138.0
[BSA] : [4a] = 1 : 3	1	230/355	249.8
	2	280/355	300.8
	a	290/295	167.9
[BSA] : [4b] = 1 : 3	1	230/355	199.8
	2	280/355	242.6
	a	290/295	140.7
[BSA] : [4c] = 1 : 3	1	230/355	254.6
	2	280/355	338.3
	a	290/295	210.9
[BSA] : [4d] = 1 : 3	1	230/355	263.8
	2	280/355	331.4
	a	290/295	155.5
[BSA] : [genistein] = 1 : 3	1	230/355	259.2
	2	280/355	333.2
	a	290/295	159.6

#### 3.4.4 Circular dichroism spectroscopy

**Fig. 8:**
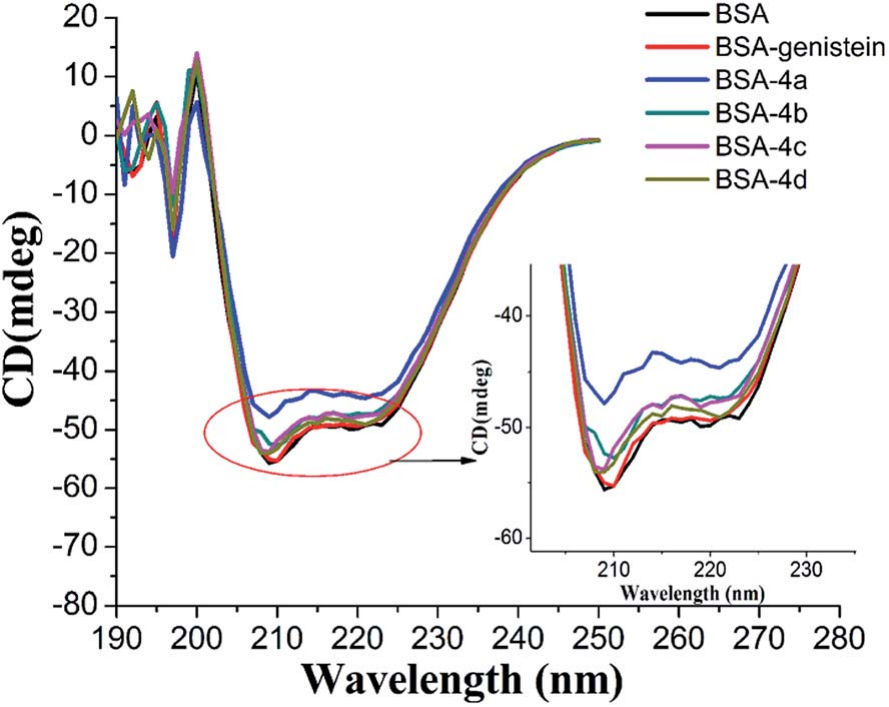
CD spectra of BSA with and without genistein, 4a, 4b, 4c, and 4d.The concentrations of BSA and all compounds were 4 μM.

CD spectra are commonly applied to study the secondary structure changes in protein during ligand–protein binding. In our study, the CD spectra of BSA with and without compounds 4a–4d were measured and shown in Fig. 8. Obviously, there were two negative absorption bands at nearby 208 nm and 222 nm, respectively. These are the characteristic peaks of the α-helical of protein.^36^ As shown in Fig. 8, with the compounds added, the absorption intensity of α-helical characteristic peak decreased, which meant that the combination of compounds 4a–4d with BSA could decrease the content of α-helical. This result was in accordance with the results of UV and synchronous fluorescence experiments. From Fig. 8, we can also find out that the changes of the content of α-helical of protein induced by compounds 4a–4d were greater than the parent skeleton genistein, which further confirmed that introducing amino acid group into genistein can enhance its interaction with BSA.

### 3.5 Molecular docking

**Fig. 9:**
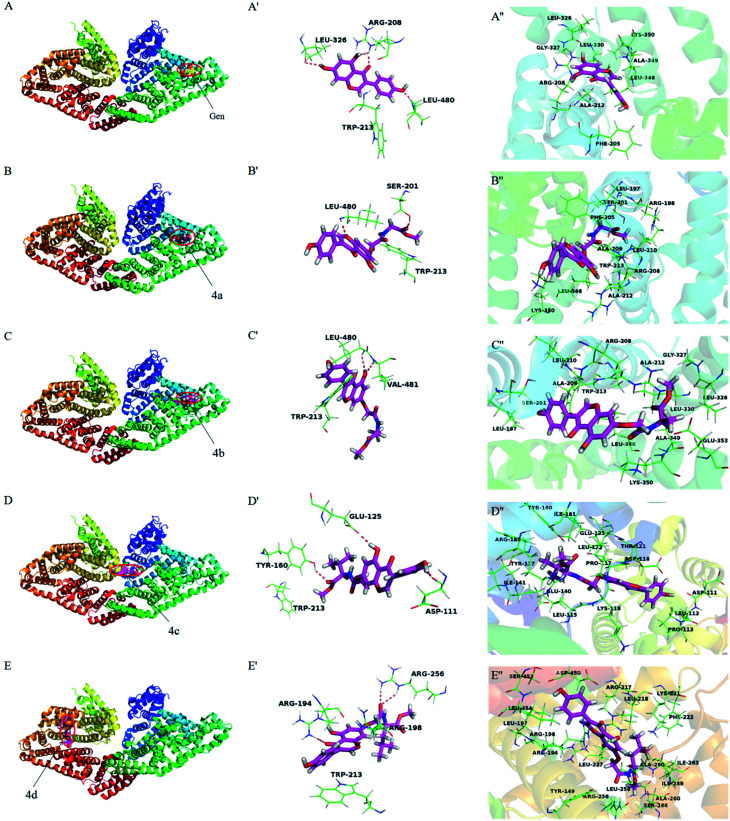
The lowest energy conformation of compounds-BSA complexes obtained from molecular docking (A–E). The hydrogen bonding interactions of genistein and compounds 4a–4d with amino acid residues of BSA (A′–E′). Amino acid residues surrounding genistein and compounds 4a–4d within 3 Å (A′′–E′′). Notes: labels of A–E correspond to genistein and compounds 4a–4d, respectively.

Molecular docking has become a popular method to predict the interaction between ligands and proteins. The conformations of BSA-compounds complexes with the lowest energy were represented in Fig. 9. Obviously, genistein and compounds 4a–4c inserted into the sub-domain IIA of BSA, while compound 4d was apt to bind on site IIIA. There were hydrogen bondings interactions of genistein and compounds 4a–4d with some amino acid residues in the binding pocket. For instance, genistein formed three hydrogen bondings with Arg208, Leu326 and Leu480 and compound 4c formed three hydrogen bondings with Tyr160, Asp111 and Glu125. The binding pockets formed by the interactions of genistein and compounds 4a–4d with BSA were close to Trp213 residue, and this was the reason for the fluorescence quenching of these ligands binding to BSA. Besides, the distance between compounds 4a–4b and Trp 213 was closer compared to genistein and 4c–4d, which indicating that the abilities of 4a–4b to quench the fluorescence of BSA were greater than that of genistein and 4c–4d. The amide bond and ester groups of 4a–4d inserted into hydrophobic loop created by Arg198, Ser201, Ala209, Phe205, Leu210 residues, indicating that van der Waals force was also involved. Furthermore, there were many hydrophobic residues surrounding genistein and compounds 4a–4d, which suggested that hydrophobic interactions may exist. For example, compound 4b was surrounded by Leu197, Ala209, Ala212, Leu210, Leu326, Trp213, Gly327, Lys350 residues. From the above analyses, it can be speculated that the priority driving force of the interaction between genistein, compounds 4a–4d and BSA was hydrogen bonding, and van der Waals force and hydrophobic interaction played a minor part, which was consistent with the results of Section 3.3.3.

## 4. Conclusion

Herein, four novel genistein amino acid derivatives 4a–4d were synthesized and their cytotoxicity activities were evaluated. The results showed that compound 4b with alanine chain showed best activity against MGC-803 cell lines with IC_50_ value of 12.08 μM. Then the interactions between compounds 4a–4d and BSA were explored by spectral techniques and molecular docking. Fluorescence quenching experiments proved that all five compounds genistein, 4a–4d quenched the fluorescence of BSA through static quenching mechanism. The thermodynamic data suggested that hydrogen bonding interaction played a major role in the bonding process, van der Waals and hydrophobic force were also involved. Synchronous and 3D fluorescence spectra indicated combination of compounds and BSA could change the microenvironment of endogenous fluorophore (Trp and Typ). The CD spectra demonstrated the content of α-helical in the BSA secondary structure was obviously decreased in the presence of compounds 4a–4d. In conclusion, introducing the amino acid group into genistein can not only improve its antitumor activity, but also enhance its binding affinity with BSA. These data were of great significance for future studies on the structural modification and pharmacokinetics of genistein *in vivo*.

## Conflicts of interest

There are no conflicts to declare.

## Supplementary Material

RA-008-C8RA06691D-s001
