# Supplementary material for: Synthesis of novel genistein amino acid derivatives and investigation on their interactions with bovine serum albumin by spectroscopy and molecular docking
Source: RSC Adv. 2018 Sep 5;8(54):31201–12. doi: 10.1039/c8ra06691d (PMC9085648; doi:10.1039/c8ra06691d)
Supplement: RA-008-C8RA06691D-s001 [file RA-008-C8RA06691D-s001.pdf]

Electronic Supplementary Material (ESI) for RSC Advances.
This journal is © The Royal Society of Chemistry 2018

1. Synthesis of genistein amino acid methyl ester derivatives (Fig. 2)

Ethyl 2-((5-hydroxy-3-(4-hydroxyphenyl)-4-oxo-4H-chromen-7-yl) oxy) acetate (**2**)

To a solution of genistein (7.4 mmol) in DMF was added KOH (7.4 mmol) and the mixture was stirred at 60°C for 1 h. Next, ethyl bromoacetate (14.8 mmol) and KI (0.74 mmol) were added and the solution was stirred for 5 h. After cooling, the mixture was poured into 500 ml distilled water. Yellow precipitate formed and was filtered from the solution. The precipitate was dissolved and purified by column chromatography on silica gel (DCM: acetone = 40:1, V/V) to afford compound **2** as yellowish solid in 53% yield.

2-((5-hydroxy-3-(4-hydroxyphenyl)-4-oxo-4H-chromen-7-yl)oxy)acetic acid (**3**)

To a solution of compound **2** (1.0 mmol) in methanol (1.0 ml) and water (4.0 ml) was added KOH (1.0 mol/L) to adjust pH to 12-13. The mixture was stirred at 60°C for 1 h. After cooling, the reaction mixture was treated with aqueous H_2SO_4 (0.5 mol/L) to adjust pH to 2-3. White precipitate formed and was filtered from the solution. The precipitate was dried in vacuo to afford **3** as a white solid.

General procedures for preparation of **4a–4d**

To a solution of compound **3** (1.0 mmol) in DMF (15.0 ml) was added EDCI·HCl (3.0 mmol) and HOBt·H_2O (3.0 mmol) at 0°C. After 4 h, DIPEA (3.0 mmol), DMAP (2.0 mmol) and amino acid methyl esters (3.0 mmol) were added. The reaction mixture was stirred at room temperature for 24 h and then poured into 200 ml distilled water. Yellow or white precipitate formed and was filtered from the solution. The precipitate was dissolved and purified by column chromatography on silica gel (PE: EtOAc = 1:2, V/V).

Methyl (2-((5-hydroxy-3-(4-hydroxyphenyl)-4-oxo-4H-chromen-7-yl) oxy) acetyl) glycinate (**4a**)

White solid, 45% yield. ^{1}H NMR (400 MHz, DMSO-d_6) δ 12.96 (s, 1H), 9.62 (s, 1H), 8.64 (t, *J* = 5.9 Hz, 1H), 8.43 (s, 1H), 7.40 (d, *J* = 8.5 Hz, 2H), 6.83 (d, *J* = 8.5 Hz, 2H), 6.68 (d, *J* = 2.1 Hz, 1H), 6.47 (d, *J* = 2.1 Hz, 1H), 4.72 (s, 2H), 3.94 (d, *J* = 5.9

Hz, 2H), 3.65 (s, 3H).^{13}C NMR (101 MHz, DMSO-d_6) δ 180.9, 170.5, 168. 0, 163.8, 162.2, 158.0, 157.7, 155.0, 130.6, 123.1, 121.5, 115.6, 106.3, 99.2, 93.7, 67.5, 52.3, 40.9. ESI-MS: calculated $C_{20}H_{17}ON_8$ [M+H]$^+$: 400.1033; Found 400.1942.

Methyl (2-((5-hydroxy-3-(4-hydroxyphenyl)-4-oxo-4H-chromen-7-yl) oxy) acetyl) alaninate (**4b**)

White solid, 46% yield. ^{1}H NMR (400 MHz, DMSO-d_6) δ 12.96 (s, 1H), 9.61 (s, 1H), 8.62 (d, *J* = 7.3 Hz, 1H), 8.42 (s, 1H), 7.40 (d, *J* = 8.6 Hz, 2H), 6.84 (d, *J* = 8.6 Hz, 2H), 6.65 (d, *J* = 2.2 Hz, 1H), 6.45 (d, *J* = 2.3 Hz, 1H), 4.75 – 4.64 (m, 2H), 4.40 (p, *J* = 7.2 Hz, 1H), 3.65 (s, 3H), 1.35 (d, *J* = 7.3 Hz, 3H).^{13}C NMR (101 MHz, DMSO-d_6) δ 180.9, 173.1, 167.3, 164.0, 162.1, 158.0, 157.8, 155.0, 130.6, 123.0, 121.5, 115.6, 106.2, 99.2, 93.7, 67.4, 52.4, 47.9, 17.3. ESI-MS: calculated $C_{21}H_{19}ON_8$ [M+H] $^+$: 414.1190; Found 414.2010.

Methyl (2-((5-hydroxy-3-(4-hydroxyphenyl)-4-oxo-4H-chromen-7-yl) oxy) acetyl) valinate (**4c**)

Yellowish solid, 56% yield. ^{1}H NMR (500 MHz, DMSO) δ 12.95 (s, 1H), 9.61 (s, 1H), 8.46 (d, J = 8.2 Hz, 1H), 8.41 (s, 1H), 7.40 (d, J = 8.6 Hz, 2H), 6.84 (d, J = 8.6 Hz, 2H), 6.62 (d, J = 2.2 Hz, 1H), 6.43 (d, J = 2.2 Hz, 1H), 4.77 (q, J = 14.8 Hz, 2H), 4.26 (dd, J = 8.1, 6.4 Hz, 1H), 3.67 (s, 3H), 2.12 (dq, J = 13.5, 6.7 Hz, 1H), 0.91 (t, J = 7.1 Hz, 6H).^{13}C NMR (126 MHz, DMSO-d6) δ 180.9, 172.2, 167.7, 164.2, 162.2, 158.0, 157.8, 154.9, 130.6, 123.0, 121.5, 115.6, 106.2, 99.0, 93.6, 67.2, 57.8, 52.3, 30.3, 19.4, 18.7. ESI-MS: calculated $C_{23}H_{23}ON_8$ [M+Na]$^+$: 464.1322; Found 464.1317.

Methyl (2-((5-hydroxy-3-(4-hydroxyphenyl)-4-oxo-4H-chromen-7-yl) oxy) acetyl) leucinate (**4d**)

Yellowish solid, 62% yield. ^{1}H NMR (400 MHz, DMSO) δ 12.95 (s, 1H), 9.61 (s, 1H), 8.57 (d, J = 7.5 Hz, 1H), 8.43 (s, 1H), 7.40 (d, J = 8.0 Hz, 2H), 6.83 (d, J = 8.0 Hz, 2H), 6.64 (s, 1H), 6.44 (s, 1H), 4.72 (q, J = 15.0 Hz, 2H), 4.38 (s, 1H), 3.64 (s, 3H), 1.68 – 1.49 (m, 3H), 0.87 (dd, J = 20.5, 5.4 Hz, 6H).^{13}C NMR (101 MHz, DMSO-d6) δ 180.9, 173.0, 167.6, 164.0, 162.2, 158.0, 157.8, 155.0, 130.6, 123.0, 121.5, 115.6, 106.2, 99.2, 93.7, 67.4, 52.4, 50.5, 24.7, 23.2, 21.6. ESI-MS: calculated $C_{24}H_{25}ON_8$ [M+H]$^+$:456.1659; Found 456.2265.
